# The prevention of diabetic foot ulceration: how biomechanical research informs clinical practice

**DOI:** 10.1590/bjpt-rbf.2014.0195

**Published:** 2016-11-16

**Authors:** Frank E. DiLiberto, Judith F. Baumhauer, Deborah A. Nawoczenski

**Affiliations:** 1 Department of Physical Therapy, Rosalind Franklin University of Medicine & Science, North Chicago, IL, USA; 2 Department of Orthopaedics, School of Medicine and Dentistry, University of Rochester, Rochester, NY, USA

**Keywords:** diabetes mellitus, foot ulcer, foot biomechanics, multi-segment foot modeling

## Abstract

**Background:**

Implementation of interprofessional clinical guidelines for the prevention of neuropathic diabetic foot ulceration has demonstrated positive effects regarding ulceration and amputation rates. Current foot care recommendations are primarily based on research regarding the prevention of ulcer recurrence and focused on reducing the magnitude of plantar stress (pressure overload). Yet, foot ulceration remains to be a prevalent and debilitating consequence of Diabetes Mellitus. There is limited evidence targeting the prevention of first-time ulceration, and there is a need to consider additional factors of plantar stress to supplement current guidelines.

**Objectives:**

The first purpose of this article is to discuss the biomechanical theory underpinning diabetic foot ulcerations and illustrate how plantar tissue underloading may precede overloading and breakdown. The second purpose of this commentary is to discuss how advances in biomechanical foot modeling can inform clinical practice in the prevention of first-time ulceration.

**Discussion:**

Research demonstrates that progressive weight-bearing activity programs to address the frequency of plantar stress and avoid underloading do not increase ulceration risk. Multi-segment foot modeling studies indicate that dynamic foot function of the midfoot and forefoot is compromised in people with diabetes. Emerging research demonstrates that implementation of foot-specific exercises may positively influence dynamic foot function and improve plantar stress in people with diabetes.

**Conclusion:**

Continued work is needed to determine how to best design and integrate activity recommendations and foot-specific exercise programs into the current interprofessional paradigm for the prevention of first-time ulceration in people with Diabetes Mellitus.

## BULLET POINTS

- Exercise is often overlooked in current ulcer prevention guidelines.
- Abnormal plantar loading and foot function may contribute to ulceration.
- Progressive weight-bearing programs can be considered for ulcer prevention.
- Foot-specific exercises may improve foot function and ulcer prevention.
- Continued study may endorse inclusion of exercise into current guidelines.

## Introduction

Approximately one out of eleven adults, equating to 415 million people worldwide, have diagnosed or undiagnosed Diabetes Mellitus (DM)^1^. The prevalence of DM and the corresponding health and socioeconomic burden are expected to get worse^1^ ^,^ ^2^. Moreover, effort to halt the rise of DM is a global target of the World Heath Organization^2^.

Among the numerous multi-system health consequences of DM, foot ulceration is an all too common problem. Lifetime prevalence estimations of foot ulceration in people with DM are as high as 25%, with a yearly incidence rate of 2-4%^3^ ^-^ ^7^. While both vascular and neuropathic processes contribute to tissue breakdown, the majority of foot ulcers are neuropathic in nature^8^ ^-^ ^10^. Importantly, foot ulceration is associated with decreased mobility and quality of life, ulcer recurrence, infection, and subsequent lower limb amputation^11^. Despite these staggering data, many of the adverse sequelae of DM, including neuropathic foot ulceration, are considered preventable^2^ ^,^ ^11^.

Clinical guidelines have been developed to direct treatment and prevention strategies for foot ulceration, and are regularly updated based on current evidence^12^ ^,^ ^13^. An interprofessional approach is advocated and many components of ulcer prevention guidelines (regular foot care, avoidance of barefoot walking) are highly recommended. Nevertheless, the quality of evidence to support many components of prevention guideline recommendations range from low to moderate^14^. Although the most substantial evidence supports recommendations for therapeutic footwear intervention, the supporting evidence is primarily based on research focused on the prevention of ulcer *recurrence* ^14^ ^,^ ^15^. Ulcer recurrence is clearly a critical issue, as current re-ulceration rates approach 40%^16^; however, the high ulcer recurrence rates also create paramount concern regarding the need to prevent *first-time* ulceration. Yet, the evidence to guide preventative efforts of first-time ulceration is strikingly limited^15^. Accordingly, recommendations for the prevention of first-time ulceration are primarily based on biomechanical theory and extract outcomes of research surrounding the prevention of recurrent ulceration. Limited evidence specifically targeting first-time ulcer prevention impedes a clinician’s ability to advise people with DM with no history of ulceration, but with risk of tissue breakdown.

Biomechanical research has been an integral component underlying our understanding of tissue breakdown and in the development of ulcer prevention guidelines. For example, the use of specialized footwear and/or insoles to relieve zones of high plantar pressure is founded on the biomechanical theory that plantar tissue overload creates tissue breakdown. While off-loading footwear is vital to ulcer healing and important to the prevention of ulcer recurrence, it is less clear how and when to apply the tissue overloading principle to the prevention of first-time ulceration. Recent evidence suggests that too much off-loading, as measured by decreased weight-bearing activity frequency, may be counterproductive in ulcer prevention efforts^16^ ^-^ ^18^. Additionally, research supporting the relationship between abnormal dynamic foot function and first-time ulceration risk is growing. Specifically, research utilizing advanced multi-segment foot modeling approaches has identified changes in kinematic and kinetic performance that are present before ulceration and deformity^19^ ^-^ ^22^. The presence and persistence of abnormal dynamic foot function prior to tissue breakdown may be an important component to target in the prevention of first-time foot ulceration. While not yet incorporated into clinical guidelines, intervention studies targeting activity frequency and dynamic foot function are beginning to establish clinically informative findings^23^ ^-^ ^25^.

The purposes of this article are to present the contemporary biomechanical theory underpinning diabetic foot ulcerations and to discuss how advances in research and biomechanical foot modeling can inform and influence clinical practice in the prevention of first-time ulceration.

## Contemporary theory

Neuropathic foot ulceration begins with insufficient blood glucose control, and it is a combination of intrinsic and extrinsic elements that beset the foot of an individual with DM. Peripheral neuropathy coupled with the external stress of weight bearing has been recognized historically as the primary pathway to neuropathic ulceration^26^ ^-^ ^28^. This framework is supported by investigations identifying elevated HbA1c levels, sensory neuropathy, and elevated plantar pressures as key factors precipitating tissue breakdown^8^ ^,^ ^29^ ^-^ ^31^. In this traditional paradigm, plantar breakdown is the consequence of tissue *overloading* via momentary high stress or the accumulation of undetected and repeated low to moderate stress on an area of the plantar neuropathic foot^26^. Animal model investigations illustrate how the *repetition* of mechanical loading is as important as the *magnitude* of loading and how repeated exposure to episodes of *stress* lowers the threshold of tissue injury^28^ ^,^ ^32^ ^-^ ^35^.

Therefore, external mechanical stress is a composite value that includes direction of load application, time (repetition, duration, and rate), and magnitude (force/area)^36^; however, it is not only tissue overloading that propagates tissue breakdown. Mechanical *underloading* may precede mechanical overloading of plantar tissue. People with DM and peripheral neuropathy (DMPN) have reduced or variable weight-bearing activity prior to ulceration^17^ ^,^ ^18^. This finding implies that a reduction in weight-bearing activity fosters a physiological environment that breeds integumentary tissue atrophy and tissue that is less resistant to stress^36^ ^,^ ^37^. Thus, the threshold for tissue injury may be lowered to a point where an event as simple as an uncharacteristically longer walk or a change in footwear initiates tissue damage. These findings emphasize that tolerance to stress is a dynamic characteristic that is contingent on prior tissue conditioning.

## Current clinical guidelines

Current ulcer prevention guidelines advocate for an interprofessional approach that includes physicians, nurses, physical therapists, orthotists, caregivers, and patients. A comprehensive strategy, including regular glucose monitoring, patient education, daily foot inspection, regular foot screenings and care, and footwear modification, is recommended^12^ ^,^ ^14^. The reader is referred to Bus et al.^14^ (International Working Group on the Diabetic Foot) and the Clinical Guidelines of the American Diabetes Association^12^ for additional guideline information. Importantly, research demonstrates that an interprofessional approach can improve ulceration and amputation rates^15^ ^,^ ^38^ ^-^ ^40^.

Contemporary recommendations for footwear intervention primarily ascribe to the overloading theory of ulceration and remain an important aspect of ulcer prevention. Footwear interventions aimed at reducing the *magnitude* of plantar loading (pressure) are commonly combined with reduced weight-bearing activity to decrease the *repetition* of loading. Evidence suggests footwear intervention should aim for a pressure relief target value of 30% to reduce and redistribute plantar pressure and thus mitigate the potential for tissue breakdown^14^. Areas of callusing and foot deformity are particularly important to address with footwear and insole interventions. Mueller et al.^41^ demonstrated that structural factors (i.e., toe deformity) can account for up to 53% of the variance in forefoot plantar pressure in people with DMPN. Therefore, best practice includes addressing structural deformity and zones of elevated pressure in a patient-specific manner *and* the assessment of the pressure-relieving effects of the prescribed custom footwear/insoles. Further, footwear intervention is contingent on patient adherence, as adherence below 80% negatively affects the footwear efficacy^11^ ^,^ ^14^ ^,^ ^42^.

In a systematic review, van Netten et al.^15^ reported that footwear intervention (typically employed in combination with an interprofessional approach) improves *re-ulceration* rates. While the positive effects of footwear intervention are clear, differences in study designs and footwear/insole strategies (over-the-counter vs. custom) have created different results across studies. Nevertheless, a recent randomized clinical trial (RCT) that followed best practice guidelines demonstrated that people with DMPN had less ulcer recurrence when wearing custom footwear with monitored pressure relief (<200 kPA or 25% reduction at targeted forefoot/midfoot sites) versus a custom footwear only group^42^. Importantly, significance at 18-month follow up (group difference of 22% in ulcer recurrence) was only found in the subset of subjects who registered high adherence. This study highlights the importance of both pressure relief assessment and patient adherence.

Recommendations for prevention of first-time ulceration also rely on the overloading paradigm previously described. Rizzo et al.^43^ conducted an RCT investigating the effect of custom footwear and insoles versus standard care upon ulceration rates in a sample that primarily included people with DMPN and no history of ulceration. The custom footwear group demonstrated significantly less ulceration incidence at 1, 3, and 5-year time points (5-year: 23.5% vs. 70%). These findings support the advantages of early custom footwear intervention; however, research on the efficacy of footwear intervention in first-time ulceration rates remains limited^15^. Further, while risk factors for ulceration have been established (i.e., loss of protective sensation)^30^ ^,^ ^44^, there is still a need to establish clinically accessible biomarkers that are predictive of imminent tissue damage. Establishment of more robust foot screening practices would assist clinicians in determining the critical time point for offloading footwear interventions^11^ ^,^ ^14^.

While the need for more research regarding footwear intervention and foot screening practices remains important, additional factors that may also prevent first-time tissue breakdown should be considered. These factors include the frequency of weight-bearing activity and the quality of dynamic foot function. Weight-bearing activity frequency is an important consideration as it pertains to plantar tissue tolerance to stress. Rather than reducing weight-bearing activity prior to tissue damage in the at-risk patient, maintenance or progression of weight-bearing activity (i.e., walking programs) may help avoid tissue underloading. An understanding of dynamic foot function and how the foot may be internally stressed during each step of every day in a patient with DMPN is equally important. Abnormal stress secondary to altered dynamic foot function from neuropathic tissue changes may expedite deformity and plantar tissue damage. With continued research focused on weight-bearing activity and dynamic foot function, interventions to modify these factors may advance and warrant inclusion in clinical guidelines.

## Advancing clinical guidelines

### Weight-bearing activity

While exercise (including weight-bearing activity) is recommended to improve glycemic control, current guidelines are vague regarding how clinicians should dose and promote exercise in people with DM who are at risk for ulceration. Further, activity increase in people with DM is complicated by co-morbid medical conditions. Yet, equally important is the idea that plantar tissue may already be deconditioned prior to first-time ulceration. Research demonstrates that too much off-loading during preventative care may potentially *increase* the risk for future ulceration^17^ ^,^ ^18^ ^,^ ^37^.

Efforts to increase weight-bearing activity and promote plantar tissue resilience and glycemic control in people with DMPN are promising regarding tissue breakdown. Two RCTs demonstrate that ulcer rates do not increase following an intervention to increase weight-bearing activity^23^ ^,^ ^24^. LeMaster et al.^23^ evaluated the effect of a weight-bearing activity program in people with DMPN and demonstrated no difference in ulceration rates between the intervention group and control group at one-year follow-up. Mueller et al.^24^ evaluated the effect of weight-bearing versus non-weight-bearing exercise on measures of activity and HbA1C levels in people with DMPN. The weight-bearing group demonstrated improvements in daily step count and there was no difference in ulceration rates between groups. Weight-bearing activity in these studies involved graduated walking programs and balance and leg strengthening exercises. Importantly, appropriate footwear and pre- and post-exercise foot inspections were included in both studies. Weight-bearing activity can be viewed as an adjunct to current interprofessional ulcer prevention guidelines for people with DMPN.

At present, conscientious patient instruction should include a program of regular daily weight-bearing activity in an attempt to improve glycemic control and potentially maintain plantar tissue integrity^23^ ^,^ ^45^; however, a weight-bearing activity exercise program to promote tissue integrity and *reduce* ulceration risk has yet to be delineated. A possible barrier to the development of such a protocol is the lack of an established biomarker (beyond patient daily foot inspection and regular clinical evaluations) to guide the assessment of plantar tissue integrity. The inability to definitively assess tissue integrity and adjust activity dosage in a patient-specific manner likely precludes inclusion of weight-bearing activity regimens in current preventative guidelines. For this reason, it is relevant to consider not only how often the foot is stressed during weight-bearing activity, but also *how* it is stressed.

### Dynamic foot function

**Figure 1:**
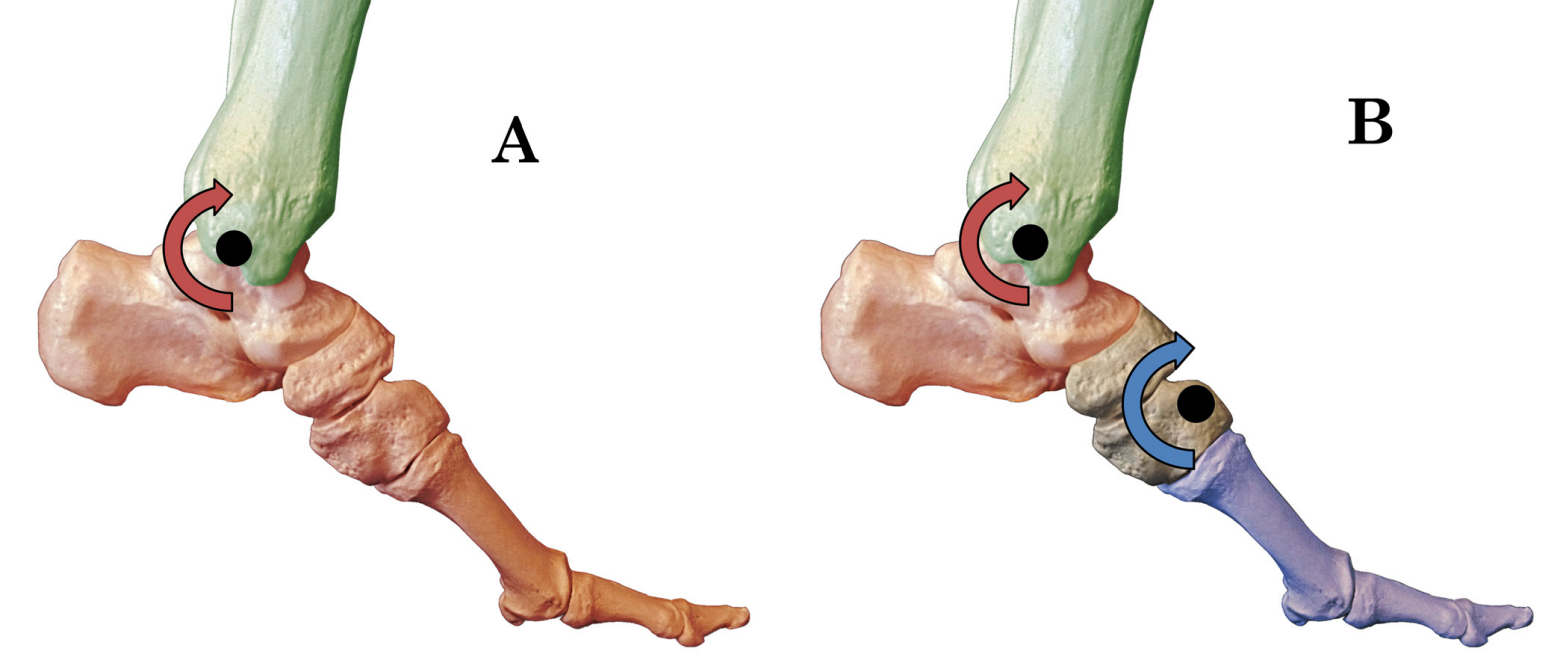
Illustrations of the osseous foot during push-off of gait. (A) The single-segment modeling approach that treats the whole foot (red) as a rigid body, moving about the tibia (green). In this model, motion and power can be measured only at the ankle; (B) A multi-segment modeling approach depicting the forefoot (blue), rearfoot (red), and tibia (green) as the three rigid body segments. In this model, motion and power can be measured at the midfoot and ankle.

In-vivo assessment of dynamic foot function fosters hypotheses generation about how internal structures are performing and consequently stressed during weight-bearing activity. Additionally, dynamic foot function represents the behavior of the foot during the time period when the primary extrinsic stimulus of ulceration occurs (i.e., walking). Dynamic foot function can be assessed using single- or multi-segment modeling approaches (Figure 1). Single-segment foot modeling is an approach that models the whole foot as a rigid body moving about the ankle joint on the tibia. While this approach provides a general overview of foot function, it may mask regional characteristics of foot function. The evolution of multi-segment foot modeling approaches has allowed for the assessment of foot function that is more congruent with the anatomical reality of the foot. The evaluation of three or more segments using multi-segment modeling approaches has advanced knowledge about both normal and pathological foot function^46^ ^-^ ^48^.

Single-segment modeling investigations of foot biomechanics in people with DMPN have produced mixed results regarding factors potentially linked to tissue breakdown. There have been variable findings regarding passive ankle joint mobility, ankle motion during walking, and forefoot plantar pressure^49^ ^-^ ^51^. Rao et al.^50^ found no association between passive and dynamic ankle motion (during walking) and no group differences in dynamic ankle motion or plantar pressures between people with DMPN and healthy controls^50^, whereas Sacco et al.^51^ found decreased ankle motion during gait in people with DMPN compared to controls. One possible explanation for between-study differences may be that midfoot and forefoot motion are embedded, rather than specifically represented, in ankle kinematic findings when using a single-segment model.

Studies employing multi-segment foot modeling approaches have identified changes in kinematics during walking in people with DM and DMPN and in people with DMPN with a history of ulceration^20^ ^,^ ^22^ ^,^ ^46^ ^,^ ^52^ ^-^ ^54^. Although differences in modeling approaches and study samples limit cross-study comparisons, these studies demonstrate that motion at multiple foot segments in people with DMPN is different from healthy controls. In general, there is a reduced amount of motion present at the midfoot/forefoot and metatarsophalangeal joints^19^ ^,^ ^20^ ^,^ ^52^. DiLiberto et al.^20^ demonstrated differences in individual metatarsal motions within the forefoot region, which may contribute to the location of elevated plantar pressures. Further, Rao et al.^55^ associated reductions in frontal plane calcaneus range of motion (ROM), as well as 1^st^ metatarsal and lateral forefoot sagittal plane ROM, during walking to elevated forefoot plantar pressures in people with DMPN. Changes in DMPN multi-segment kinematics are also present during higher-level tasks. Hastings et al.^56^ demonstrated decreased forefoot relative to rearfoot plantarflexion (lower medial longitudinal arch) during a heel raise task in people with DMPN and medial column deformity, in comparison to controls.

The use of multi-segment models in diabetic foot research demonstrates how more specific modeling approaches can better link regions of foot dysfunction to regions of diabetic foot pathology (toe and midfoot deformity, forefoot ulceration, and Charcot arthropathy); however, relationships between foot kinematics and plantar pressures are commonly generated from data collected during separate walking trials. Giacomozzi et al.^57^ developed an integrated instrumentation approach (compounded pressure platform and force plate) that allows for the simultaneous collection of kinematics, ground reaction forces, and plantar pressure during the same step of a walking trial. This approach has been applied to healthy individuals and people with DM to better align regional plantar pressure measurements to specific joint motions^22^ ^,^ ^47^ ^,^ ^53^. With continued application, integrated approaches may further elucidate linkages between multi-segment kinematics, plantar loading, and potentially multi-joint powers in people with DMPN.

Application of single- and multi-segment modeling approaches has also advanced our understanding of kinetic performance (i.e., joint power) in people with DMPN. Studies using single-segment foot models demonstrate a reduction in peak ankle plantarflexion power during walking in people with DMPN, as compared to healthy controls^49^ ^,^ ^55^; however, assessment of ankle power using a single-segment approach overestimates ankle power and precludes evaluation of midfoot kinetic performance^58^. Accordingly, multi-segment assessment of *both* ankle and midfoot power may better reflect the degree of internal stresses, particularly at the midfoot, during functional tasks in people with DMPN. DiLiberto et al.^21^ evaluated ankle and midfoot power in people with DMPN. When compared to matched controls, people with DMPN exhibited greater power absorption and less power generation during walking at both the ankle and midfoot. These findings implicate deficiencies in muscle performance and additional active supporting mechanisms at the ankle *and* midfoot as factors of abnormal dynamic foot function in people with DMPN. Specifically, the reduced active mechanism support at the arch raises questions regarding abnormal internal stresses on passive structures at both regions of the foot. The cumulative effect of this kinetic pattern may contribute to the development and/or progression of midfoot deformity^21^.

Changes in DMPN dynamic foot function have been attributed to neuropathic tissue changes such as decreased tissue extensibility (i.e., joint capsules and plantar fascia) and muscle atrophy/fatty infiltration. Recent research specifically supports the relationship between neuropathic muscle changes, multi-segment foot kinematics, and deformity in people with DMPN. Hastings et al.^59^ demonstrated that increased intrinsic muscle fat content and decreased ankle plantarflexion strength predict altered forefoot to rearfoot kinematics during a heel raise task. This study also found that altered intrinsic muscle volumes and posterior tibialis tendon volumes were related to medial column alignment (deformity). Similarly, Cheuy et al.^60^ ^,^ ^61^ demonstrated associations between intrinsic muscle fatty infiltration, changes in ankle and metatarsophalangeal joint motion during active dorsiflexion, and severity of toe deformity. Given the link between structural deformity, plantar pressure, and tissue breakdown, interventions targeting muscle function are worthy of exploration in future research. Moreover, since motor neuropathy may precede sensory neuropathy^62^, earlier intervention is potentially better.

## Intervention studies on dynamic foot function

There is increasing focus on how to design intervention strategies to improve dynamic foot function in people with DM who are at risk for ulceration^63^. Foot-specific interventions target abnormal multi-segment kinematics and kinetics and aim to improve or slow the decline in muscle function and joint mobility associated with neuropathic processes. The operating hypothesis is that improved foot function will result in better force transfer and pressure redistribution on the plantar foot^63^.

Some studies have demonstrated promising biomechanical results by incorporating foot-specific exercises in people with DMPN^25^ ^,^ ^64^ ^-^ ^66^. Common elements of foot-specific interventions include toe/forefoot ROM, ankle and subtalar ROM, and intrinsic and extrinsic foot muscle strengthening. Sartor et al.^25^ conducted an RCT in people with DMPN. The 12-week intervention was comprised of foot-specific exercise, functional training, and gait training. While most improvements were not maintained at the 24-week follow-up, improvements in plantar pressure distribution and foot biomechanics (roll over) were noted at 12 weeks in the intervention group. Further, strength changes, assessed manually by a physical therapist, showed intrinsic and extrinsic muscle strength gains in the intervention group (in contrast with a decline in the DMPN control group who received standard care). These findings provide supportive rationale for future studies on how foot-specific exercises may improve ulcer prevention efforts. Future research on the design of foot-specific exercise programs is important to determine the most effective exercises, the appropriate stage in the neuropathic process for exercise implementation, and carryover.

## Clinical implications and conclusions

Preservation of plantar tissue integrity and foot function for the prevention of first-time neuropathic foot ulceration is a challenging endeavor for people with DMPN and their care providers. The effectiveness of preventative interventions is predicated on blood glucose control, the advancement of the neuropathic process, and plantar tissue integrity. It is imperative for clinicians to address the interplay between the magnitude (pressure), frequency (weight-bearing activity), and quality (dynamic foot function) of foot stress. One of the most difficult challenges is to determine how to strike an effective balance between too much (overloading) and too little (underloading) stress. When deciding between parameters of loading, the clinician is advised that the threshold for tissue damage is contingent on both the current state of the foot tissue and how it will be stressed in the future.

While there is room for improvement regarding foot-screening procedures to best evaluate tissue integrity and predict injury, recent research offers insight that may augment current clinical guidelines. In addition to the interprofessional approach and foot care practices described previously, advising patients on interventions to address the frequency and quality of stress should be considered. A progressive and well-monitored weight-bearing activity regimen that includes walking, balance, and leg strengthening can positively influence patient health and function. Activity regimens not only promote glycemic control, potentially slowing the effects of neuropathy, but also possibly decrease the potential of plantar tissue underloading. Further, multi-segment modeling investigations suggest that addressing dynamic foot function is an additional supplement for the prevention of first-time ulceration. Specifically, assessment of forefoot and midfoot mobility, as well as assessment of intrinsic and extrinsic muscle strength should be part of routine foot screening examinations. Examination findings should guide clinical decisions for implementation of progressive exercise programs to improve dynamic foot function or direct patients to other appropriate interventions/care providers (i.e., footwear). Continued work is needed to determine how to best design and integrate activity recommendations and foot-specific exercise programs into the current interprofessional paradigm for the prevention of first-time ulceration in people with Diabetes Mellitus.
